# Why move abroad? Factors influencing migration intentions of final year students of health-related disciplines in Nigeria

**DOI:** 10.1186/s12909-023-04683-6

**Published:** 2023-10-10

**Authors:** Temitope Olumuyiwa Ojo, Blessing Pelumi Oladejo, Bolade Kehinde Afolabi, Ayomide Damilola Osungbade, Princely Chukwunenye Anyanwu, Ikeme Shaibu-Ekha

**Affiliations:** 1 Department of Community Health, Obafemi Awolowo University, Ile-Ife, +234 8035798224 Osun State Nigeria; 2 Department of Community Health, Obafemi Awolowo University Teaching Hospitals Complex, Ile Ife, Nigeria

**Keywords:** Migration intention, Human Resources for Health, Health-related disciplines, Final year students, Nigeria

## Abstract

**Background:**

Limited human resource for health may impede the attainment of health-related sustainable development goals in low-income countries. This study aims to identify migration factors among final-year students of health-related disciplines at a Nigerian university, reflecting trends in Nigeria and sub-Saharan African countries.

**Methods:**

A cross-sectional study was conducted using a semi-structured, self-administered questionnaire to collect data from 402 final-year students of Medicine/Dentistry, Nursing, Pharmacy and Occupational therapy Physiotherapy at Obafemi Awolowo University, Ile Ife. Univariate, bivariate and multivariate data analysis were conducted and a p-value < 0.05 was taken as statistically significant.

**Results:**

The mean age of the respondents was 24.3 ± 2.3 years. Most (326; 81.1%) respondents had intentions to migrate and majority (216; 53.7%) of respondents had an unfavourable attitude towards practising in Nigeria. Students of Nursing constitute the highest proportion (68; 91.9%) of those willing to migrate (p = 0.009). The common preferred destinations for those who intend to migrate were the United Kingdom (84; 25.8%), Canada (81; 24.8%), and the United States of America (68; 20.9%). Respondents who had favourable attitude towards practicing abroad (AO.R: 2.9; 95% C.I 1.6–5.2; p = 0.001) were three times more likely to have migration intentions compared with those who had an unfavourable attitude towards practicing abroad, while the odds for those who had favourable attitude towards practicing in Nigeria (AO.R: 0.4; 95% C.I 0.2–0.7; p = 0.002) was two times less than those who had an unfavourable attitude towards practice in Nigeria. Respondents who desire specialist training (AO.R: 3.0; 95% C.I 1.7–5.4; p < 0.001) were three times more likely to have intention to migrate abroad when compared to those who were undecided or had no desire to pursue specialist training.

**Conclusion:**

Most respondents had the intention to migrate abroad after graduation and this could be attributed to the desire for specialist training and their attitude towards practising in Nigeria. Interventions aimed at improving specialist training in Nigeria and incentivizing health care practice may reduce migration trends among Nigeria’s health professionals in training.

**Supplementary Information:**

The online version contains supplementary material available at 10.1186/s12909-023-04683-6.

## Background

Health professionals’ migration is an increasing challenge in many low- and middle-income countries. For instance, Africa bears 24% of the disease burden globally and has only 2% of the global health workforce [1]. This upsurge in migration is largely driven by the increasing disparity between the demand and availability of health professionals in developed nations [2].

The Global Health Report (WHO, 2006) issued a distinct call to action within the international community to tackle the estimated shortage of over 4.3 million health personnel collectively referred to as Human Resources for Health (HRH). This shortage poses significant challenges with far-reaching implications linked to migration [3]. The World Health Organization (WHO) proposed that the Sustainable Development Goal (SDG) benchmark for health workers per population should be 4.45 doctors, nurses, and midwives per 1000 individuals [4].

Globally, the enhancement of the health workforce (HWF) faces challenges, particularly evident in Sub-Saharan Africa, where the health worker density has declined to 1.55 per 1000 population [4]. A significant contributor to this decline is migration from low and middle-income countries to more promising prospects abroad, driven by a combination of push and pull factors [5]. Push factors such as poor working conditions, inadequate remuneration, and limited career development prospects push health professionals to seek better opportunities elsewhere (5). Conversely, developed nations attract health workers with improved working conditions, higher pay, and greater career prospects, serving as pull factors that entice professionals to pursue opportunities overseas. The allure of these pull factors leads to a reduction in the health worker density in their home countries, posing challenges to the regional health workforce in Africa and beyond (5).

This situation has negatively impacted the availability and quality of healthcare services and delivery to people due to the depletion of Human Resources for Health [6]. Moreover, the morale of the remaining workforce who are now overworked remains dampened [2]. Although new medical schools and numerous schools for other health-related disciplines were recently established in Africa, they only serve to produce more health professionals for greener pastures [7].

Recently, the Nigerian health sector experienced increased migration of health professionals, to foreign lands. A study carried out among doctors undergoing residency and internship programmes in a tertiary hospital in Ekiti state, Nigeria, revealed that Resident doctors undergoing specialist training in many public tertiary hospitals constitute a significant proportion of physicians who had left or have the intention to leave Nigeria for other nations of the world [2]. Among those with intentions to go abroad, 70% of the respondents had taken steps towards realizing their plans [2]. About 86% of all physicians from the sub-Saharan African (SSA) region employed in the United States are from only three countries; Nigeria (40%), South Africa and Ghana, of which 79% are trained at only ten medical schools [8].

The phenomenon of migration is also applicable to final-year students of health-related disciplines all over the world who form a ready pool of potential labour migrants. There is a growing preference for migration abroad among graduates and students of health disciplines reported from past studies in Nigeria [7] and Morocco [9]. However, in a study conducted by Silvestri et al. comparing medical and Nursing students in a cross-sectional survey in Asia and Africa [10], international careers were anticipated by 24% of all medical students and 36% of all Nursing students as a motivating factor.

Estimates of nurse migration are more diverse with increasing number of nurses trained in Sub-Saharan Africa now practicing abroad. [11] Similarly, the brain drain potential of pharmacy students in Nigeria has been evaluated across three Nigerian universities [12]. Majority of the survey respondents had intentions of emigrating from Nigeria and students less than 25 years were more likely to have migration intentions when compared to older students [12]. Reasons adduced for migration intentions among students of health-related disciplines are quite varied and include a search for better salary pay, better training, better working system, and better quality of life [2, 13, 14].

There is a significant amount of research highlighting the migration intention of medical professionals and that the COVID pandemic increased the odds of the willingness healthcare professionals to migrate [2, 9]. However, despite the multiplicity of data on the migration intent of healthcare workers and its effect on the health care system [15], existing studies have ignored the migration intention of students of health-related disciplines. More individuals are becoming disillusioned with the way of living in Nigeria and are currently seeking a way out of the system, with students in health-related disciplines beginning to see their career path as a means to an end, and the younger generation seeking to carve out a career in health-related professions overseas [16].

The migration of health professionals in Nigeria and many other sub-Saharan African countries presents a growing challenge, leading to a significant decrease in the health workforce density [5, 12]. Various factors, including cultural, structural, educational, financial, and socio-political aspects, contribute to this trend, varying depending on the specific nation [5, 9, 11]. However, a common consequence of these factors is a compromised health service system, which negatively impacts the availability and quality of healthcare services in Africa [11]. As a result, Human Resources for Health (HRH) progressively becomes depleted, posing significant challenges in efforts to strengthen the health workforce. Therefore, understanding the underlying reasons behind this migration trend in these countries is essential for developing effective interventions and addressing the implications it presents [5, 11, 12].

According to the WHO Workforce 2030, several policies to manage human resources for health were proposed including; policies on production affecting the education sector, policies to address inflows and outflows of the labour market, maldistribution and inefficiencies of the labour market [17]. Migration policies in receiving countries have been found to be instrumental in stemming health professional migration. It is obvious that these strategies have yielded limited results. Therefore, understanding why healthcare workers or trainees intend to migrate is essential to effective human resource for health (HRH) management in resource-limited countries [18]. Hence, this study assessed the migration intentions of final-year students in health-related disciplines. The study findings may provide information needed to draw up interventions aimed at addressing migration trends among Human Resources for Health (HRH) in Nigeria and many other Sub-Saharan African countries.

## Methods

### Study location

The study was conducted at Obafemi Awolowo University, Ile-Ife, Osun State, Nigeria. Obafemi Awolowo University is one of the largest public universities located in Nigeria. It was established in 1962 and currently has about 35,000 students and over 12,000 staff [19]. The University has two colleges namely the College of Health Sciences and the Postgraduate College: 13 faculties and over hundred departments. Students of health-related disciplines are students from the departments of Medicine/Dentistry, Nursing, Pharmacy, Occupational therapy and Physiotherapy. All these departments run some courses in common which makes them related, such courses include; Human anatomy, Physiology, Biochemistry in addition to the preclinical exposure to basic science courses such Physics, Chemistry, and Biology. Upon graduation, these students have the option to either practice with their basic qualifications or choose to pursue further specialization in their respective fields.

### Study design

A descriptive cross-sectional design was employed for this study.

### Study population

The study population were final year students in health-related disciplines, which included Medicine/Dentistry, Nursing, Pharmacy, Occupational therapy and Physiotherapy at Obafemi Awolowo University, Ile-Ife. However, final year students who were acutely ill, or on leave of absence were excluded. Study was conducted between January and February 2023.

### Sample size determination

The minimum sample size was determined using Leslie Fisher’s formula for calculating sample size for simple proportions in a known finite population. The total number of students of health-related disciplines who were in their final year at the time of study was 504. The sample size was calculated with Zα, standard normal deviate corresponding to 95% confidence level, proportion of doctors at Ekiti state teaching hospital, Nigeria who were considering migration (74.2%) obtained from study by Akinwumi et al. (2), and corrected for anticipated non-response rate of 20% because the period of study was within the examination period for students in some of these departments, the minimum sample size calculated for the study was rounded up to 400.

### Sampling technique

Students were divided into strata based on their individual departments. The departmental strata were Medicine/Dentistry, Nursing, Pharmacy, Occupational therapy/ Physiotherapy. Thereafter, a sample proportionate to size was calculated for each stratum. Using the class list as a sample frame, students were selected from each departmental stratum via a table of random numbers.

### Outcome and explanatory variables

The primary outcome measure for this study was the migration intention of final year students of health-related disciplines categorized into “yes” and “no”. Explanatory variables include sociodemographic characteristics, intention to pursue specialist training, attitudes towards practice in Nigeria and attitudes towards practice abroad.

### Research instrument

A semi-structured, self-administered questionnaire was used to obtain information about sociodemographic characteristics, intention to migrate, attitudes towards migration and reasons for not wanting to migrate. The study instrument was adapted from a validated questionnaire previously used to assess migration intentions of doctors in postgraduate training in Nigeria. [2].

### Data collection and analysis

Data were collected from 402 final year students of health-related disciplines at Obafemi Awolowo University. The questionnaire had the following sections: Sociodemographic characteristics, Intention to Migrate, Attitudes towards migration and Reasons for not wanting to Migrate.

Data entry and analysis were conducted using IBM Statistical Product and Service Solutions (SPSS) version 25, installed on passworded systems. The consistency of the data input was confirmed by double entry and random checking. Attitude towards practicing in Nigeria was assessed using 6 questions with responses on a 5-likert scale. Each response was scored as follows; strongly agree = 1, agree = 2, neutral = 3, disagree = 4 and strongly disagree = 5), the scores for each respondent was summed and a median score of 12 was used to categorise into unfavourable (< 12) and favourable attitude (≥ 12). In addition, attitude towards practising abroad was assessed using 6 questions with responses on a 5-likert scale. Each response was scored as follows; strongly agree = 5, agree = 4, neutral = 3, disagree = 2 and strongly disagree = 1). After summing up the total score for each respondent, a median score of 21 was used to categorise into unfavourable (< 21) and favourable attitude (≥ 21). The association between discrete variables was tested using the chi-square test and a multivariate binary logistic regression analysis with adjusted odds ratios (AOR) was done to determine the association between the dependent (intention to migrate) and independent variables. Only variables that were significant at 25% or less (p < 0.25) were included in the regression model [20]. A p-value < 0.05 was considered as statistically significant.

Ethical consideration: The ethical approval to conduct this study was obtained from the health research and ethics committee of the Institute of Public Health, Obafemi Awolowo University, Ile-Ife, Nigeria with approval number HREC: IPH/OAU/12/2137. Informed consent was obtained from all respondents. In addition, the study complied with the Declaration of Helsinki on conducting research among human subjects.

## Results

Four hundred and two questionnaires were administered to eligible respondents.

Table 1 shows the socio-demographic characteristics of the respondents. Majority of the respondents (216; 53.7%) were females and less than 25 years (247; 61.4%). Also, most of the respondents were Christians (354; 88.1%) and of Yoruba ethnicity (335; 83.3%). Almost all (382; 95%) of the respondents were single and concerning monthly stipends, majority (166; 43.35%) received less than 30,000 naira ($70) monthly.

**Table 1:** Socio-demographic characteristics of respondents

Variable	Frequency (n)	Percent (%)
**Age (years**)		
< 25	247	61.4
≥ 25	155	38.6
(Mean ± SD = 23.4 ± 2.3 years)		
**Sex**		
Male	186	46.3
Female	216	53.7
**Course of study**		
Medicine/Dentistry	125	31.1
Physiotherapy/Occupational therapy	103	25.6
Nursing	74	18.4
Pharmacy	100	24.9
**Religion**		
Christianity	354	88.1
Islam	46	11.4
Traditional	2	0.5
**Ethnicity**		
Yoruba	335	83.3
Igbo	43	10.7
Hausa	2	0.5
Others	22	5.5
**Marital status**		
Single	382	95.0
Married	20	5.0
**Spouse profession**		
Healthcare worker	7	35.0
Non-healthcare worker	13	65.0
**Academic status**		
Had 1 or more distinctions	92	22.9
Straight pass	166	41.3
Had 1 or more resits	107	26.6
Repeated a class	37	9.2
**Average monthly stipend (Naira)**		
< 30,000	225	56.0
≥ 30,000	177	44.0
Median (IQR) = 25,000 (20,000–40,000)		

The attitudes towards practice in Nigeria and migrating abroad are shown in Table 2 Most of the respondents had a favourable attitude towards migration, they viewed the working conditions and living conditions in the country as unfavourable. Of the total 402 respondents, 331(82.3%) indicated that the living condition in Nigeria was unmanageable, and another 333 (82.8%) had negative views on the working conditions.

**Table 2:** Attitude of respondents towards practice in Nigeria and migrating abroad

Variable	Strongly Agree (n%)	Agree (n%)	Neutral (n%)	Disagree (n%)	Strongly Disagree (n%)
I am not satisfied with my job prospects in Nigeria	142 (35.3)	143 (35.6)	69 (17.2)	35 (8.7)	13 (3.2)
There is little scope in Nigeria for promotion accountability & qualifications.	115 (28.6)	157 (39.1)	85 (21.1)	36 (9.0)	9 (2.2)
Living conditions in Nigeria are becoming unmanageable.	185 (46.0)	146 (36.3)	50 (12.4)	17 (4.2)	4 (1.0)
I am unhappy with the way our government treats doctors.	219 (54.5)	112 (27.9)	49 (12.2)	17 (4.2)	5 (1.2)
I am unhappy with the negative attitudes of Nigerian public/society towards doctors.	159 (39.6)	139 (34.6)	88 (21.9)	14 (3.5)	2 (0.5)
Working conditions in Nigeria make it difficult to fulfil the noble ideals of my profession.	181 (45.0)	152 (37.8)	51 (12.7)	17 (4.2)	1 (0.2)
I think I will be able to cope with working conditions abroad.	128 (31.8)	168 (41.8)	91 (22.6)	12 (3.0)	3 (0.7)
Status of doctors is higher in foreign countries than in Nigeria.	132 (32.8)	135 (33.6)	107 (26.6)	26 (6.5)	2 (0.5)
I think I will be able to adjust to social and political conditions abroad.	101 (25.1)	171 (42.5)	114 (28.4)	15 (3.7)	1 (0.2)
I think that those who migrate to foreign countries cannot hope to enjoy equal rights of citizenship.	48 (11.9)	138 (34.3)	147 (36.6)	59 (14.7)	10 (2.5)
Health professionals have to work much harder in foreign countries than in Nigeria.	62 (15.4)	145 (36.1)	129 (32.1)	58 (14.4)	8 (2.0)
Health professionals have less job security in foreign countries than in Nigeria	41 (10.2)	81 (20.1)	121 (30.1)	121 (30.1)	38 (9.5)

The proportion of respondents who intend to migrate after undergraduate studies was 326 (81.1%) as shown in Table 3. Of the total respondents, 211 (64.7%) had plans to migrate within five years of finishing medical school with 132 (40.5%) planning to finance their migration plans via family support. Also, of the 326 respondents who intend to migrate, 164 (50.3%) do not intend to return to practice in Nigeria. However, out of the 150 respondents who planned to return to Nigeria, 80 (53.3%) hope to return to Nigeria after 10 years of migrating abroad.

**Table 3:** Migration intentions of respondents and summary of attitude towards practicing abroad/ Nigeria

Variable	Frequency (n)	Percent (%)
**Intend to migrate**		
Yes	326	81.1
No	76	18.9
**Intended time of migration**		
Immediately after school	83	25.5
Within 5 years of finishing school	211	64.7
After 5 years of finishing school	32	9.8
**Proposed sources for funding migration intention**		
Personal effort	114	35.0
Family support	132	40.5
Undecided	80	24.5
**Preferred destination of respondents**		
United Kingdom	84	25.8
United States of America	68	20.9
Canada	81	24.8
Australia	22	6.7
Others	7	2.1
Undecided	64	19.6
**Currently working on migration**		
Yes	142	43.6
No	184	56.4
**Purpose of migration**		
For further specialty training	146	44.8
For practice as a health professional	133	40.8
Undecided	39	12.0
Others	8	2.5
**Studying course with the intention to migrate**		
Yes	55	16.9
No	271	83.1
**Respondents plan to return to Nigeria to practice**		
Yes	150	46.0
No	164	50.3
Undecided	12	3.7
**Intended time to return**		
Less than 5years	20	13.3
Within 5-10years	50	33.3
After 10years	80	53.3
**Intend to pursue specialist training**		
Yes	282	70.1
No/undecided	120	29.9
**Attitude towards practicing in Nigeria**		
Unfavourable attitude	216	53.7
Favourable attitude	186	46.3
**Attitude towards migrating abroad**		
Unfavourable attitude	209	52.0
Favourable attitude	193	48.0

Also from Table 3, of the 326 respondents who want to migrate, a higher proportion (84; 25.8%) intend to migrate to the United Kingdom while the other preferred countries include, Canada (81; 24.8%), the United State of America (68; 20.9%), Australia (22; 6.7%), and some few Middle Eastern countries (7; 2.1%).

Table 4 shows the association between the intention to migrate and sociodemographic characteristics of respondents. Students of Nursing sciences have the highest proportion of respondents who intend to migrate (68; 91.9%) and was statistically significant (p = 0.009). Also, a higher proportion (246; 87.2%) of students who intend to pursue specialist training have migration intention and this was also found to be statistically significant (p < 0.001). Respondents with both favourable and unfavourable attitudes towards practice in Nigeria have the intention to migrate. However, a higher proportion (87.5% ) of those who have unfavourable attitudes towards practice in Nigeria intend to migrate. (p < 0.001) Also, a higher proportion (89.6%) of those with favourable attitudes towards migrating abroad have the intention to migrate (p < 0.001).

**Table 4:** Association between intention to migrate and selected respondents’ characteristics

Variable	Intention to migrate		Statistics
	Yes N (%)	No N (%)	
**Age (years)**			
< 25	205(83.0%)	42 (17.0%)	χ^2^ = 1.511 p = 0.219
≥ 25	121 (78.1%)	34 (21.9%)	
**Sex**			
Male	145 (78.0%)	41 (22.0%)	χ^2^ = 2.223 p = 0.136
Female	181 (83.8%)	35 (16.2%)	
**Course of study**			
Medicine/ Dentistry	100 (80.0%)	25 (20.0%)	χ^2^ = 11.507 p = 0.009
Physiotherapy/Occupational therapy	86 (83.5%)	17 (16.5%)	
Nursing	68 (91.9%)	6 (8.1%)	
Pharmacy	72 (72%)	28 (28%)	
**Religion**			
Christianity	286 (80.8%)	68 (19.25%)	χ^2^ = 1.691 p = 0.429
Islam	39 (84.8%)	7 (15.2%)	
Others	1 (50%)	1 (50%)	
Average monthly stipend (Naira)			
< 30,000	180 (80.0)	45 (20.0)	χ^2^ = 0.399
≥ 30,000	146 (82.5)	31 (17.5)	p = 0.527
**Academic status in last three years**			
1 or more distinction	75 (81.5%)	17 (18.5%)	χ^2^ = 5.473 p = 0.140
Straight pass	132 (79.5%)	34 (20.5%)	
Had one or more resits	93 (86.9%)	14 (13.1%)	
Repeated a class	26 (70.3%)	11 (29.7%)	
**Intend to pursue specialist training**			
Yes	246 (87.2%)	36 (12.8%)	χ^2^ = 23.226 p = < 0.001
No	80 (66.7%)	40 (33.3%)	
**Attitude to practice in Nigeria**			
Unfavourable attitude	189 (87.5%)	27 (12.5%)	χ^2^ = 12.494 p = < 0.001
Favourable attitude	137 (73.7%)	49 (26.3%)	
**Attitude to practice abroad**			
Unfavourable attitude	153 (73.2%)	56 (26.8%)	χ^2^ = 17.671 p = < 0.001
Favourable attitude	173 (89.6%)	20 (10.4%)	

χ^2^ = chi square value, p = p-value

On multivariate analysis, respondents who had favourable attitude towards practising abroad (AO.R: 2.9; 95% C.I 1.6–5.2; p = 0.001) were three times more likely to have intentions to migrate abroad after graduation than their colleagues with unfavourable attitude. The odds for those who had favourable attitude towards practicing in Nigeria (A.O.R: 0.4; 95% C.I 0.2–0.7; p = 0.002) was two times less than those who had unfavourable attitude towards practice in Nigeria. Finally, respondents who desire specialist training (AO.R: 3.0; 95% C.I 1.7–5.4; p < 0.001) were three times more likely to have intention to migrate abroad when compared to those who were undecided or had no desire to pursue specialist training. (Table 5).

However, the association between the intention to migrate and other sociodemographic characteristics such as age, sex, religion, ethnicity, marital status, and academic status in the last three (3) years were not statistically significant.

**Table 5:** Binary logistic regression analysis of the factors influencing migration intentions of respondents

Variable	Adjusted Odds Ratio	95% Confidence Interval	p- values
**Sex of Respondents**			
Female	Ref		
Male	0.8	0.4–1.5	0.491
**Age**			
≥ 25	Ref		
< 25	1.2	0.7–2.2	0.508
**Course of Study**			
Pharmacy	Ref		
Medicine/Dentistry	1.0	0.5–2.1	0.999
Physiotherapy/Occupational therapy	1.5	0.7–3.1	0.490
Nursing	2.6	0.9–7.4	0.068
**Academic Status in last three years**			
Repeated a class	Ref		
Had One or more distinctions	1.8	0.6–4.9	0.276
Straight pass	1.4	0.5–3.6	0.476
Had One or more resits	2.6	0.9–7.2	0.074
**Intent to pursue specialist training**			
No/Undecided	Ref		
Yes	3.0	1.7–5.4	< 0.001
**Attitude towards practice in Nigeria**			
Unfavourable attitude	Ref		
Favourable attitude	0.4	0.2–0.7	0.002
**Attitude towards practice abroad**			
Unfavourable attitude	Ref		
Favourable attitude	2.9	1.6–5.2	0.001

## Discussion

The study found that the respondents had mixed views on their future career opportunities and professional practice. Most of the respondents viewed the living condition as unmanageable, and also had negative views about the working condition obtainable within the country. These findings are similar to results obtained from studies focused on migration intentions of low and middle-income countries and this has largely impacted on students in this region [5, 8, 21–23].

Most respondents had favourable attitude towards migrating abroad and this aligns with findings from past studies. For instance, a study in Ethiopia revealed that the attitude towards migration was higher among clinical (63%) and Internship (71%) students. [24]. This may suggest that students’ attitudes toward migration and practice can be influenced during their undergraduate training, and these attitudes can affect their intentions of migrating after graduation. Therefore, it is important to enhance the prospects of practice for students in training, create conducive training and working environments, and implement retention initiatives to ensure their commitment to serve their nation.

The findings of this study are relevant to other sub-Saharan African (SSA) countries like Ghana and South Africa who are grappling with substantial “brain drain” in their healthcare sectors. The migration factors identified in our study, including push factors like perceived poor working conditions in home country and pull factors like perceived better opportunities abroad, also apply to these countries as well. [25, 26] While there may be subtle differences in context between Nigeria and these other SSA countries, the insights from our study could inform targeted policies to address healthcare workforce challenges in the SSA region.

Majority (81.1%) of our respondents intend to migrate after undergraduate studies. This proportion is higher than that obtained in different parts of Africa [24, 27] and from other similar international studies [1, 28–30]. It is also higher than that obtained in a similar study done in Nigeria which revealed that 74.4% of respondents preferred to migrate abroad to specialize [7]. This tendency can likely be attributed to the economic context of the region (a low/middle-income country) and the period during which the study was conducted. The dynamics have changed in the post-COVID-19 era as a similar study done indicated that more health professionals have made up their mind about migrating abroad [2].

A sub-analysis done revealed that Nursing students comprised the highest proportion of respondents who wanted to migrate. Findings from the present study are consistent with reports from similar previous studies among nurses undergoing training programs, which revealed that most of the respondents intend to migrate [31, 32]. This may be due to the fact that nurses are in high demand globally [33] and they are able to integrate into newer environments more smoothly than other health professionals without the need to undergo numerous certification examinations.

Among the minor proportion of respondents (18.9%) who do not intend to migrate, a higher proportion have personal desires to live in the country and serve their people and nation. Hence, this study revealed that the cost of migration, cost and worries about examination, perceived racism and inability to adapt to the weather abroad were not the main reasons why respondents do not intend to migrate as identified in some other studies [34, 35] .

Similar to a previous study, the U.K. (25.8%), Canada (24.8%) and the USA (20.9%) were the top three preferred destinations of respondents wanting to leave the country [27]. This is also similar to findings from a study on the migration profile of Nigerians which showed that the U.K. (40%), Canada (17.6%) and the USA (15.7%) were the most preferred destinations [14]. These findings are also in tandem with findings from several international studies which assessed the preferred destinations of health professionals [5, 8, 9, 13, 27] This common migration pattern is indicative of strong pull factors that exist in those upper-income countries, such as high job satisfaction and better remuneration among physicians in USA and U.K, which widely varies with the relatively poor satisfaction found in our study. Furthermore, these countries, notably the UK, have demonstrated lenient immigration policies which also may facilitate subsequent migration to other nations. This factor further bolsters migration intentions [32, 36].

A study by Ossai et al. revealed that the students who have decided on a specialty of choice were more likely to emigrate after graduation as compared with those who were yet to make such decisions [7] and this was similar to the finding from our study. This phenomenon might stem from the fact that students who are keen on emigrating start strategizing during undergraduate studies, all in preparation for that specific goal.

However, our study’s findings regarding factors associated with migration factors differ from findings in few previous studies within the country. For instance, Onah et al. which revealed that the commonest reasons respondents wanted to migrate were poor remuneration, rising insecurity and inadequate diagnostic facilities. [14]. This difference may probably be due to the difference in work status with our respondents being students while their respondents were practising health professionals.

Future research should investigate determinants of migration intention among health professions students using a multi-site study approach. This approach holds promise in providing nuanced insights into the evolving intentions of students at various stages of their training. Employing a mixed methods approach in future studies will also offer a comprehensive understanding of the intricate social contexts that underlie migration intentions among health professions’ students in training.

This study had some limitations, firstly, data collection was conducted using self-administered questionnaire, potentially introducing the influence of social desirability bias. This implies that respondents may provide responses which are socially acceptable. Secondly, the study employed quantitative methods only, hence it precluded the possibly of obtaining social perspectives and nuanced insights about respondents’ migration intentions Lastly, the study was conducted in one Nigerian university, which may potentially affect generalizability.

## Conclusion

The study concludes that most respondents intended to migrate, with the United Kingdom, the United States of America, and Canada as their top preferences. Those seeking specialist training and health professions displayed a higher inclination to migrate abroad, while only a minority were willing to specialize in Nigeria due to personal desires or commitment to national service. This trend of migration intention among students of health-related disciplines has huge policy implications and could negatively impact health service delivery in the country. To address this challenge, policy recommendations include broadening postgraduate training opportunities, offering more specializations, improving welfare packages and incentives, enhancing healthcare facilities, and implementing a student loans program to encourage professionals retention in the country, ultimately bolstering the availability of Human Resources for Health (HRH) in Nigeria and many other Sub-Saharan African countries.

### Electronic supplementary material

Below is the link to the electronic supplementary material.

Supplementary Material 1

## Data Availability

Datasets supporting the findings of this study are available on request to the corresponding author.
