# Supplementary material for: Why move abroad? Factors influencing migration intentions of final year students of health-related disciplines in Nigeria
Source: BMC Med Educ. 2023 Oct 10;23:742. doi: 10.1186/s12909-023-04683-6 (PMC10563360; doi:10.1186/s12909-023-04683-6)
Supplement: Supplementary file 1 — Supplementary Material 1 [file 12909_2023_4683_MOESM1_ESM.docx]

**Why Move Abroad? Factors Influencing Migration Intentions of Final Year Students of Health-Related Disciplines in Nigeria**

Temitope Olumuyiwa Ojo^1,2^, Blessing Pelumi Oladejo^1^, Bolade Kehinde Afolabi^2^, Ayomide Damilola Osungbade^1^, Princely Chukwunenye Anyanwu^1^, Ikeme Shaibu-Ekha^1^

Affiliations

^1^Department of Community Health, Obafemi Awolowo University, Ile Ife, Nigeria

^2^Department of Community Health, Obafemi Awolowo University Teaching Hospitals Complex, Ile Ife, Nigeria

**Corresponding author**

Dr Temitope Olumuyiwa Ojo.

Department of Community Health, Obafemi Awolowo University, Ile-Ife, Osun State, Nigeria

+234 8035798224; linktopeojo@yahoo.com

**ABSTRACT**

**Background:** Limited human resource for health may impede the attainment of health-related sustainable development goals in low-income countries. This study aims to identify migration factors among final-year students of health-related disciplines at a Nigerian university, reflecting trends in Nigeria and sub-Saharan African countries.

**Methods:** A cross-sectional study was conducted using a semi-structured, self-administered questionnaire to collect data from 402 final-year students of Medicine/Dentistry, Nursing, Pharmacy, Occupational therapy, and Physiotherapy at Obafemi Awolowo University, Ile Ife. Univariate, bivariate and multivariate data analysis were conducted and a p-value <0.05 was taken as statistically significant.

**Results:** The mean age of the respondents was 24.3 ±2.3 years. Most (326; 81.1%) respondents had intentions to migrate and that majority (216; 53.7%) of respondents had an unfavourable attitude towards practising in Nigeria. Students of Nursing constitute the highest proportion (68; 91.9%) of those willing to migrate (p=0.009). The common preferred destinations for those who intend to migrate were the United Kingdom (84; 25.8%), Canada (81; 24.8%), and the United States of America (68; 20.9%). Respondents who had favourable attitude towards practicing abroad (AO.R: 2.9; 95% C.I 1.6 – 5.2; p=0.001) were three times more likely to have migration intentions compared with those who had an unfavourable attitude towards practicing abroad, while the odds for those who had favourable attitude towards practicing in Nigeria (AO.R: 0.4; 95% C.I 0.2 – 0.7; p=0.002) was two times less than those who had an unfavourable attitude towards practice in Nigeria. Respondents who desire specialist training (AO.R: 3.0; 95% C.I 1.7 – 5.4; p<0.001) were three times more likely to have intention to migrate abroad when compared to those who were undecided or had no desire to pursue specialist training.

**Conclusion:** Most respondents had the intention to migrate abroad after graduation and this could be attributed to the desire for specialist training and their attitude towards practising in Nigeria. Interventions aimed at improving specialist training in Nigeria and incentivizing health care practice may reduce migration trends among Nigeria’s health professionals in training.

**Keywords:** Migration intention, Human Resources for Health, Health-related disciplines, Final year students, Nigeria.

**BACKGROUND**

Health professionals’ migration is an increasing challenge in many low- and middle-income countries. For instance, Africa bears 24% of the disease burden globally and has only 2% of the global health workforce[1]. This upsurge in migration is largely driven by the increasing disparity between the demand and availability of health professionals in developed nations [2].

The Global Health Report (WHO, 2006) issued a distinct call to action within the international community to tackle the estimated shortage of over 4.3 million health personnel collectively referred to as Human Resources for Health (HRH). This shortage poses significant challenges with far-reaching implications linked to migration [3]. The World Health Organization (WHO) proposed that the Sustainable Development Goal (SDG) benchmark for health workers per population should be 4.45 doctors, nurses, and midwives per 1000 individuals [4].

Globally, the enhancement of the health workforce (HWF) faces challenges, particularly evident in Sub-Saharan Africa, where the health worker density has declined to 1.55 per 1000 population [4]. A significant contributor to this decline is migration from low and middle-income countries to more promising prospects abroad, driven by a combination of push and pull factors [5]. Push factors such as poor working conditions, inadequate remuneration, and limited career development prospects push health professionals to seek better opportunities elsewhere (5). Conversely, developed nations attract health workers with improved working conditions, higher pay, and greater career prospects, serving as pull factors that entice professionals to pursue opportunities overseas. The allure of these pull factors leads to a reduction in the health worker density in their home countries, posing challenges to the regional health workforce in Africa and beyond (5).

This situation has negatively impacted the availability and quality of healthcare services and delivery to people due to the depletion of Human Resources for Health [6]. Moreover, the morale of the remaining workforce who are now overworked remains dampened [2]. Although new medical schools and numerous schools for other health-related disciplines were recently established in Africa, they only serve to produce more health professionals for greener pastures [7].

Recently, the Nigerian health sector experienced increased migration of health professionals, to foreign lands. A study carried out among doctors undergoing residency and internship programmes in a tertiary hospital in Ekiti state, Nigeria, revealed that Resident doctors undergoing specialist training in many public tertiary hospitals constitute a significant proportion of physicians who had left or have the intention to leave Nigeria for other nations of the world[2]. Among those with intentions to go abroad, 70% of the respondents had taken steps towards realizing their plans[2]. About 86% of all physicians from the sub-Saharan African (SSA) region employed in the United States are from only three countries; Nigeria (40%), South Africa and Ghana, of which 79% are trained at only ten medical schools [8].

The phenomenon of migration is also applicable to final-year students of health-related disciplines all over the world who form a ready pool of potential labour migrants. There is a growing preference for migration abroad among graduates and students of health disciplines reported from past studies in Nigeria[7] and Morocco[9]. However, in a study conducted by Silvestri et. Al comparing medical and Nursing students in a cross-sectional survey in Asia and Africa[10], international careers were anticipated by 24% of all medical students and 36% of all Nursing students as a motivating factor.

Estimates of nurse migration are more diverse with increasing number of nurses trained in Sub-Saharan Africa now practicing abroad.[11] Similarly, the brain drain potential of pharmacy students in Nigeria has been evaluated across three Nigerian universities [12]. Majority of the survey respondents had intentions of emigrating from Nigeria and students less than 25 years were more likely to have migration intentions when compared to older students [12]. Reasons adduced for migration intentions among students of health-related disciplines are quite varied and include a search for better salary pay, better training, better working system, and better quality of life [2, 13, 14].

There is a significant amount of research highlighting the migration intention of medical professionals and that the COVID pandemic increased the odds of the willingness healthcare professionals to migrate[2, 9]. However, despite the multiplicity of data on the migration intent of healthcare workers and its effect on the health care system [15], existing studies have ignored the migration intention of students of health-related disciplines. More individuals are becoming disillusioned with the way of living in Nigeria and are currently seeking a way out of the system, with students in health-related disciplines beginning to see their career path as a means to an end, and the younger generation seeking to carve out a career in health-related professions overseas [16].

The migration of health professionals in Nigeria and many other sub-Saharan African countries presents a growing challenge, leading to a significant decrease in the health workforce density [5,12]. Various factors, including cultural, structural, educational, financial, and socio-political aspects, contribute to this trend, varying depending on the specific nation [5,9,11]. However, a common consequence of these factors is a compromised health service system, which negatively impacts the availability and quality of healthcare services in Africa [11]. As a result, Human Resources for Health (HRH) progressively becomes depleted, posing significant challenges in efforts to strengthen the health workforce. Therefore, understanding the underlying reasons behind this migration trend in these countries is essential for developing effective interventions and addressing the implications it presents [5,11,12].

According to the WHO Workforce 2030 , several policies to manage human resources for health were proposed including; policies on production affecting the education sector, policies to address inflows and outflows of the labour market, maldistribution and inefficiencies of the labour market [17]. Migration policies in receiving countries have been found to be instrumental in stemming health professional migration. It is obvious that these strategies have yielded limited results. Therefore, understanding why healthcare workers or trainees intend to migrate is essential to effective human resource for health (HRH) management in resource-limited countries[18]. Hence, this study assessed the migration intentions of final-year students in health-related disciplines. The study findings may provide information needed to draw up interventions aimed at addressing migration trends among Human Resources for Health (HRH) in Nigeria and many other Sub-Saharan African countries.

**METHODS**

**Study Location**

The study was conducted at Obafemi Awolowo University, Ile-Ife, Osun State, Nigeria. Obafemi Awolowo University is one of the largest public universities located in Nigeria. It was established in 1962 and currently has about 35,000 students and over 12,000 staff [19]. The University has two colleges namely the College of Health Sciences and the Postgraduate College: 13 faculties and over hundred departments. Students of health-related disciplines are students from the departments of Medicine and Surgery, Dentistry, Nursing, Pharmacy, Occupational therapy and Physiotherapy. All these departments run some courses in common which makes them related, such courses include; Human anatomy, Physiology, Biochemistry in addition to the preclinical exposure to basic science courses such Physics, Chemistry, and Biology. Upon graduation, these students have the option to either practice with their basic qualifications or choose to pursue further specialization in their respective fields.

**Study Design:** A descriptive cross-sectional design was employed for this study.

**Study Population**

The study population were final year students in health-related disciplines, which included Medicine and Surgery, Dentistry, Nursing, Pharmacy, Occupational therapy and Physiotherapy at Obafemi Awolowo University, Ile-Ife. However, final year students who were acutely ill, or on leave of absence were excluded. Study was conducted between January and February 2023.

**Sample size determination**

The minimum sample size was determined using Leslie Fisher’s formula for calculating sample size for simple proportions in a known finite population. The total number of students of health-related disciplines who were in their final year at the time of study was 504. The sample size was calculated with Zα, standard normal deviate corresponding to 95% confidence level, proportion of doctors at Ekiti state teaching hospital, Nigeria who were considering migration (74.2%) obtained from study by Akinwumi et. Al. (2), and corrected for anticipated non-response rate of 20% because the period of study was within the examination period for students in some of these departments, the minimum sample size calculated for the study was rounded up to 400.

**Sampling Technique**

Students were divided into strata based on their individual departments. The five departmental strata were Medicine and Surgery, Dentistry, Nursing, Pharmacy, Occupational therapy and Physiotherapy. Thereafter, a sample proportionate to size was calculated for each stratum. Using the class list as a sample frame, students were selected from each departmental stratum via a table of random numbers.

**Outcome and explanatory variables**

The primary outcome measure for this study was the migration intention of final year students of health-related disciplines categorized into “yes” and “no”. Explanatory variables include sociodemographic characteristics, intention to pursue specialist training, attitudes towards practice in Nigeria and attitudes towards practice abroad.

**Research Instrument**

A semi-structured, self-administered questionnaire was used to obtain information about sociodemographic characteristics, intention to migrate, attitudes towards migration and reasons for not wanting to migrate. The study instrument was adapted from a validated questionnaire previously used to assess migration intentions of doctors in postgraduate training in Nigeria.[2]

**Data collection and analysis**

Data were collected from 402 final year students of health-related disciplines at Obafemi Awolowo University. The questionnaire had the following sections: Sociodemographic characteristics, Intention to Migrate, Attitudes towards migration and Reasons for not wanting to Migrate.

Data entry and analysis were conducted using IBM Statistical Product and Service Solutions (SPSS) version 25, installed on passworded systems. The consistency of the data input was confirmed by double entry and random checking. Attitude towards practicing in Nigeria was assessed using 6 questions with responses on a 5-likert scale. Each response was scored as follows; strongly agree=1 , agree=2, neutral=3, disagree=4 and strongly disagree=5) , the scores for each respondent was summed and a median score of 12 was used to categorise into unfavourable (<12) and favourable attitude (≥12). In addition, attitude towards practising abroad was assessed using 6 questions with responses on a 5-likert scale. Each response was scored as follows; strongly agree=5 , agree=4, neutral=3, disagree=2 and strongly disagree=1). After summing up the total score for each respondent, a median score of 21 was used to categorise into unfavourable (<21) and favourable attitude (≥21). The association between discrete variables was tested using the chi-square test and a multivariate binary logistic regression analysis with adjusted odds ratios (AOR) was done to determine the association between the dependent (intention to migrate) and independent variables. Only variables that were significant at 25% or less (p<0.25) were included in the regression model[20]. A p-value <0.05 was considered as statistically significant.

Ethical consideration: The ethical approval to conduct this study was obtained from the health research and ethics committee of the Institute of Public Health, Obafemi Awolowo University, Ile-Ife, Nigeria with approval number HREC: IPH/OAU/12/2137. Informed consent was obtained from all respondents. In addition, the study complied with the Declaration of Helsinki on conducting research among human subjects.

**RESULTS**

Four hundred and two questionnaires were administered to eligible respondents.

Table 1 shows the socio-demographic characteristics of the respondents. Majority of the respondents (216; 53.7%) were females and less than 25 years (247; 61.4%). Also, most of the respondents were Christians (354; 88.1%) and of Yoruba ethnicity (335; 83.3%). Almost all (382; 95%) of the respondents were single and concerning monthly stipends, majority (166; 43.35%) received less than 30,000 naira ($70) monthly

The attitudes towards practice in Nigeria and migrating abroad are shown in Table 2 Most of the respondents had a favourable attitude towards migration, they viewed the working conditions and living conditions in the country as unfavourable. Of the total 402 respondents, 331(82.3%) indicated that the living condition in Nigeria was unmanageable, and another 333 (82.8%) had negative views on the working conditions.

| **Variable** | **Frequency (n)** | **Percent (%)** |
| --- | --- | --- |
| **Age (years**) |  |  |
| <25 | 247 | 61.4 |
| ≥25 | 155 | 38.6 |
| (Mean ±SD = 23.4±2.3 years) |  |  |
| **Sex** |  |  |
| Male | 186 | 46.3 |
| Female | 216 | 53.7 |
| **Course of study** |  |  |
| Medicine/Dentistry | 125 | 31.1 |
| Physiotherapy/Occupational therapy | 103 | 25.6 |
| Nursing | 74 | 18.4 |
| Pharmacy | 100 | 24.9 |
| **Religion** |  |  |
| Christianity | 354 | 88.1 |
| Islam | 46 | 11.4 |
| Traditional | 2 | 0.5 |
| **Ethnicity** |  |  |
| Yoruba | 335 | 83.3 |
| Igbo | 43 | 10.7 |
| Hausa | 2 | 0.5 |
| Others | 22 | 5.5 |
| **Marital status** |  |  |
| Single | 382 | 95.0 |
| Married | 20 | 5.0 |
| **Spouse profession** |  |  |
| Healthcare worker | 7 | 35.0 |
| Non-healthcare worker | 13 | 65.0 |
| **Academic status** |  |  |
| Had 1 or more distinctions | 92 | 22.9 |
| Straight pass | 166 | 41.3 |
| Had 1 or more resits | 107 | 26.6 |
| Repeated a class | 37 | 9.2 |
| **Average monthly stipend (Naira)** |  |  |
| <30,000 | 225 | 56.0 |
| ≥30,000 | 177 | 44.0 |
| Median (IQR) = 25,000 (20,000-40,000) |  |  |

Table 1. Socio-demographic characteristics of respondents


Table 2 Attitude of respondents towards practice in Nigeria and migrating abroad

| **Variable** | **Strongly Agree (n%)** | **Agree (n%)** | **Neutral (n%)** | **Disagree (n%)** | **Strongly Disagree (n%)** |
| --- | --- | --- | --- | --- | --- |
| I am not satisfied with my job prospects in Nigeria | 142 (35.3) | 143 (35.6) | 69 (17.2) | 35 (8.7) | 13 (3.2) |
| There is little scope in Nigeria for promotion accountability & qualifications. | 115 (28.6) | 157 (39.1) | 85 (21.1) | 36 (9.0) | 9 (2.2) |
| Living conditions in Nigeria are becoming unmanageable. | 185 (46.0) | 146 (36.3) | 50 (12.4) | 17 (4.2) | 4 (1.0) |
| I am unhappy with the way our government treats doctors. | 219 (54.5) | 112 (27.9) | 49 (12.2) | 17 (4.2) | 5 (1.2) |
| I am unhappy with the negative attitudes of Nigerian public/society towards doctors. | 159 (39.6) | 139 (34.6) | 88 (21.9) | 14 (3.5) | 2 (0.5) |
| Working conditions in Nigeria make it difficult to fulfil the noble ideals of my profession. | 181 (45.0) | 152 (37.8) | 51 (12.7) | 17 (4.2) | 1 (0.2) |
| I think I will be able to cope with working conditions abroad. | 128 (31.8) | 168 (41.8) | 91 (22.6) | 12 (3.0) | 3 (0.7) |
| Status of doctors is higher in foreign countries than in Nigeria. | 132 (32.8) | 135 (33.6) | 107 (26.6) | 26 (6.5) | 2 (0.5) |
| I think I will be able to adjust to social and political conditions abroad. | 101 (25.1) | 171 (42.5) | 114 (28.4) | 15 (3.7) | 1 (0.2) |
| I think that those who migrate to foreign countries cannot hope to enjoy equal rights of citizenship. | 48 (11.9) | 138 (34.3) | 147 (36.6) | 59 (14.7) | 10 (2.5) |
| Health professionals have to work much harder in foreign countries than in Nigeria. | 62 (15.4) | 145 (36.1) | 129 (32.1) | 58 (14.4) | 8 (2.0) |
| Health professionals have less job security in foreign countries than in Nigeria | 41 (10.2) | 81 (20.1) | 121 (30.1) | 121 (30.1) | 38 (9.5) |

The proportion of respondents who intend to migrate after undergraduate studies was 326 (81.1%) as shown in Table 3. Of the total respondents, 211 (64.7%) had plans to migrate within five years of finishing medical school with 132 (40.5%) planning to finance their migration plans via family support. Also, of the 326 respondents who intend to migrate, 164 (50.3%) do not intend to return to practice in Nigeria. However, out of the 150 respondents who planned to return to Nigeria, 80 (53.3%) hope to return to Nigeria after 10 years of migrating abroad.

Table 3 Migration intention of respondents and summary of attitude towards practicing abroad/ Nigeria

| **Variable** | **Frequency (n)** | **Percent (%)** |
| --- | --- | --- |
| **Intend to migrate** |  |  |
| Yes | 326 | 81.1 |
| No | 76 | 18.9 |
| **Intended time of migration** |  |  |
| Immediately after school | 83 | 25.5 |
| Within 5 years of finishing medical school | 211 | 64.7 |
| After 5 years of finishing school | 32 | 9.8 |
| **Proposed sources for funding migration intention** |  |  |
| Personal effort | 114 | 35.0 |
| Family support | 132 | 40.5 |
| Undecided | 80 | 24.5 |
| **Preferred destination of respondents** |  | |
| United Kingdom | 84 | 25.8 |
| United States of America | 68 | 20.9 |
| Canada | 81 | 24.8 |
| Australia | 22 | 6.7 |
| Others | 7 | 2.1 |
| Undecided | 64 | 19.6 |
| **Currently working on migration** |  |  |
| Yes | 142 | 43.6 |
| No | 184 | 56.4 |
| **Purpose of migration** |  |  |
| For further specialty training | 146 | 44.8 |
| For practice as a health professional | 133 | 40.8 |
| Undecided | 39 | 12.0 |
| Others | 8 | 2.5 |
| **Studying course with the intention to migrate** |  |  |
| Yes | 55 | 16.9 |
| No | 271 | 83.1 |
| **Respondents plan to return to Nigeria to practice** |  |  |
| Yes | 150 | 46.0 |
| No | 164 | 50.3 |
| Undecided | 12 | 3.7 |
| **Intended time to return** |  |  |
| Less than 5years | 20 | 13.3 |
| Within 5-10years | 50 | 33.3 |
| After 10years | 80 | 53.3 |
| **Intend to pursue specialist training** |  |  |
| Yes | 282 | 70.1 |
| No/undecided | 120 | 29.9 |
| **Attitude towards practicing in Nigeria** |  |  |
| Unfavourable attitude | 216 | 53.7 |
| Favourable attitude | 186 | 46.3 |
| **Attitude towards migrating abroad** |  |  |
| Unfavourable attitude | 209 | 52.0 |
| Favourable attitude | 193 | 48.0 |

Also from Table 3, of the 326 respondents who want to migrate, a higher proportion (84; 25.8%) intend to migrate to the United Kingdom while the other preferred countries include, Canada (81; 24.8%), the United State of America (68; 20.9%), Australia (22; 6.7%), and some few Middle Eastern countries (7; 2.1%).

Table 4 shows the association between the intention to migrate and sociodemographic characteristics of respondents. Students of Nursing sciences have the highest proportion of respondents who intend to migrate (68; 91.9%) and was statistically significant (p=0.009). Also, a higher proportion (246; 87.2%) of students who intend to pursue specialist training have migration intention and this was also found to be statistically significant (p<0.001). Respondents with both favourable and unfavourable attitudes towards practice in Nigeria have the intention to migrate. However, a higher proportion (87.5% ) of those who have unfavourable attitudes towards practice in Nigeria intend to migrate. (p<0.001) Also, a higher proportion (89.6%) of those with favourable attitudes towards migrating abroad have the intention to migrate (p <0.001)

However, the association between the intention to migrate and other sociodemographic characteristics such as age, sex, religion, ethnicity, marital status, and academic status in the last three (3) years were not statistically significant.

Table 4 Association between intention to migrate and selected respondents’ characteristics

| **Variable** | **Intention to migrate** | | **Statistics** |
| --- | --- | --- | --- |
|  | **Yes**  **N (%)** | **No**  **N (%)** |  |
| **Age (years)** | | | |
| <25 | 205(83.0%) | 42 (17.0%) | χ^2^ = 1.511  p= 0.219 |
| ≥25 | 121 (78.1%) | 34 (21.9%) |  |
| **Sex** | | | |
| Male | 145 (78.0%) | 41 (22.0%) | χ^2^ = 2.223  p= 0.136 |
| Female | 181 (83.8%) | 35 (16.2%) |  |
| **Course of study** | | | |
| Medicine/Dentistry | 100 (80.0%) | 25 (20.0%) | χ^2^ = 11.507  p= 0.009 |
| Physiotherapy/Occupational therapy | 86 (83.5%) | 17 (16.5%) |  |
| Nursing | 68 (91.9%) | 6 (8.1%) |  |
| Pharmacy | 72 (72%) | 28 (28%) |  |
| **Religion** | | | |
| Christianity | 286 (80.8%) | 68 (19.25%) | χ^2^ = 1.691  p= 0.429 |
| Islam | 39 (84.8%) | 7 (15.2%) |  |
| Others | 1 (50%) | 1 (50%) |  |
| Average monthly stipend (Naira) |  |  |  |
| <30,000 | 180 (80.0) | 45 (20.0) | χ^2^ = 0.399 |
| ≥30,000 | 146 (82.5) | 31 (17.5) | p= 0.527 |
| **Academic status in last three years** | | | |
| 1 or more distinction | 75 (81.5%) | 17 (18.5%) | χ^2^= 5.473  p= 0.140 |
| Straight pass | 132 (79.5%) | 34 (20.5%) |  |
| Had one or more resits | 93 (86.9%) | 14 (13.1%) |  |
| Repeated a class | 26 (70.3%) | 11 (29.7%) |  |
| **Intend to pursue specialist training** | | | |
| Yes | 246 (87.2%) | 36 (12.8%) | χ^2^ = 23.226  p= <0.001 |
| No | 80 (66.7%) | 40 (33.3%) |  |
| **Attitude to practice in Nigeria** |  |  |  |
| Unfavourable attitude | 189 (87.5%) | 27 (12.5%) | χ^2^ = 12.494  p= <0.001 |
| Favourable attitude | 137 (73.7%) | 49 (26.3%) |  |
| **Attitude to practice abroad** |  |  |  |
| Unfavourable attitude | 153 (73.2%) | 56 (26.8%) | χ^2^= 17.671  p= <0.001 |
| Favourable attitude | 173 (89.6%) | 20 (10.4%) |  |

χ^2^ = chi square value, p= p-value

On multivariate analysis, respondents who had favourable attitude towards practising abroad (AO.R: 2.9; 95% C.I 1.6 – 5.2; p=0.001) were three times more likely to have intentions to migrate abroad after graduation than their colleagues with unfavourable attitude. The odds for those who had favourable attitude towards practicing in Nigeria (A.O.R: 0.4; 95% C.I 0.2 – 0.7; p=0.002) was two times less than those who had unfavourable attitude towards practice in Nigeria. Finally, respondents who desire specialist training (AO.R: 3.0; 95% C.I 1.7 – 5.4; p<0.001) were three times more likely to have intention to migrate abroad when compared to those who were undecided or had no desire to pursue specialist training. (Table 5)

Table 5 Binary logistic regression analysis of the factors influencing migration intentions of respondents

| **Variable** | **Adjusted Odds Ratio** | **95% Confidence Interval** | **p- values** |
| --- | --- | --- | --- |
| **Sex of Respondents** |  |  |  |
| Female | Ref |  |  |
| Male | 0.8 | 0.4-1.5 | 0.491 |
| **Age** |  |  |  |
| ≥25 | Ref |  |  |
| <25 | 1.2 | 0.7-2.2 | 0.508 |
| **Course of Study** |  |  |  |
| Pharmacy | Ref |  |  |
| Medicine/Dentistry | 1.0 | 0.5-2.1 | 0.999 |
| Physiotherapy/Occupational therapy | 1.5 | 0.7-3.1 | 0.490 |
| Nursing | 2.6 | 0.9-7.4 | 0.068 |
| **Academic Status in last three years** |  |  |  |
| Repeated a class | Ref |  |  |
| Had One or more distinctions | 1.8 | 0.6-4.9 | 0.276 |
| Straight pass | 1.4 | 0.5-3.6 | 0.476 |
| Had One or more resits | 2.6 | 0.9-7.2 | 0.074 |
| **Intent to pursue specialist training** |  |  |  |
| No/Undecided | Ref |  |  |
| Yes | 3.0 | 1.7-5.4 | <0.001 |
| **Attitude towards practice in Nigeria** |  |  |  |
| Unfavourable attitude | Ref |  |  |
| Favourable attitude | 0.4 | 0.2-0.7 | 0.002 |
| **Attitude towards practice abroad** |  |  |  |
| Unfavourable attitude | Ref |  |  |
| Favourable attitude | 2.9 | 1.6-5.2 | 0.001 |

**DISCUSSION**

The study found that the respondents had mixed views on their future career opportunities and professional practice**.**  Most of the respondents viewed the living condition as unmanageable, and also had negative views about the working condition obtainable within the country. These findings are similar to results obtained from studies focused on migration intentions of low and middle-income countries and this has largely impacted on students in this region[5, 8, 21–23]

Most respondents had favourable attitude towards migrating abroad and this aligns with findings from past studies. For instance, a study in Ethiopia revealed that the attitude towards migration was higher among clinical (63%) and Internship (71%) students.[24]. This may suggest that students’ attitudes toward migration and practice can be influenced during their undergraduate training, and these attitudes can affect their intentions of migrating after graduation. Therefore, it is important to enhance the prospects of practice for students in training, create conducive training and working environments, and implement retention initiatives to ensure their commitment to serve their nation.

The findings of this study are relevant to other sub-Saharan African (SSA) countries like Ghana and South Africa who are grappling with substantial "brain drain" in their healthcare sectors. The migration factors identified in our study, including push factors like perceived poor working conditions in home country and pull factors like perceived better opportunities abroad, also apply to these countries as well.[25, 26] While there may be subtle differences in context between Nigeria and these other SSA countries, the insights from our study could inform targeted policies to address healthcare workforce challenges in the SSA region.

Majority (81.1%) of our respondents intend to migrate after undergraduate studies. This proportion is higher than that obtained in different parts of Africa [24, 27] and from other similar international studies [1, 28–30]. It is also higher than that obtained in a similar study done in Nigeria which revealed that 74.4% of respondents preferred to migrate abroad to specialize [7]. This tendency can likely be attributed to the economic context of the region (a low/middle-income country) and the period during which the study was conducted. The dynamics have changed in the post-COVID-19 era as a similar study done indicated that more health professionals have made up their mind about migrating abroad [2].

A sub-analysis done revealed that Nursing students comprised the highest proportion of respondents who wanted to migrate. Findings from the present study are consistent with reports from similar previous studies among nurses undergoing training programs, which revealed that most of the respondents intend to migrate[31, 32]. This may be due to the fact that nurses are in high demand globally[33] and they are able to integrate into newer environments more smoothly than other health professionals without the need to undergo numerous certification examinations.

Among the minor proportion of respondents (18.9%) who do not intend to migrate, a higher proportion have personal desires to live in the country and serve their people and nation. Hence, this study revealed that the cost of migration, cost and worries about examination, perceived racism and inability to adapt to the weather abroad were not the main reasons why respondents do not intend to migrate as identified in some other studies[34, 35] .

Similar to a previous study, the U.K. (25.8%), Canada (24.8%) and the USA (20.9%) were the top three preferred destinations of respondents wanting to leave the country [27]. This is also similar to findings from a study on the migration profile of Nigerians which showed that the U.K. (40%), Canada (17.6%) and the Usha (15.7%) were the most preferred destinations [14]. These findings are also in tandem with findings from several international studies which assessed the preferred destinations of health professionals[5, 8, 9, 13, 27] This common migration pattern is indicative of strong pull factors that exist in those upper-income countries, such as high job satisfaction and better remuneration among physicians in USA and U.K, which widely varies with the relatively poor satisfaction found in our study. Furthermore, these countries, notably the UK, have demonstrated lenient immigration policies which also may facilitate subsequent migration to other nations. This factor further bolsters migration intentions [32, 36].

A study by Ossai et al revealed that the students who have decided on specialty of choice were more likely to emigrate after graduation as compared with those who were yet to make such decisions[7] and this was similar to the finding from our study. This phenomenon might stem from the fact that students who are keen on emigrating start strategizing during undergraduate studies, all in preparation for that specific goal.

However, our study's findings regarding factors associated with migration factors differ from findings in few previous studies within the country. For instance, Onah et al which revealed that the commonest reasons respondents wanted to migrate were poor remuneration, rising insecurity and inadequate diagnostic facilities.[14] This difference may probably be due to the difference in work status with our respondents being students while their respondents were practising health professionals.

Future research should investigate determinants of migration intention among health professions students using a multi-site study approach. This approach holds promise in providing nuanced insights into the evolving intentions of students at various stages of their training. Employing a mixed methods approach in future studies will also offer a comprehensive understanding of the intricate social contexts that underlie migration intentions among health professions’ students in training.

This study had some limitations, firstly, data collection was conducted using self-administered questionnaire, potentially introducing the influence of social desirability bias. This implies that respondents may provide responses which are socially acceptable. Secondly, the study employed quantitative methods only, hence it precluded the possibly of obtaining social perspectives and nuanced insights about respondents’ migration intentions Lastly, the study was conducted in one Nigerian university, which may potentially affect generalizability.

**CONCLUSION**

The study concludes that most respondents intended to migrate, with the United Kingdom, the United States of America, and Canada as their top preferences. Those seeking specialist training and health professions displayed a higher inclination to migrate abroad, while only a minority were willing to specialize in Nigeria due to personal desires or commitment to national service. This trend of migration intention among students of health-related disciplines has huge policy implications and could negatively impact health service delivery in the country. To address this challenge, policy recommendations include broadening postgraduate training opportunities, offering more specializations, improving welfare packages and incentives, enhancing healthcare facilities, and implementing a student loans program to encourage professionals retention in the country, ultimately bolstering the availability of Human Resources for Health (HRH) in Nigeria and many other Sub-Saharan African countries.

**DECLARATION**

**Ethics Approval and Consent to Participate**

Ethical approval was obtained from the Health Research and Ethics Committee, Institute of Public Health, College of Health Sciences, Obafemi Awolowo University, Ile- Ife with HREC no IPH/OAU/12/2137. All respondents gave voluntary informed consent after being informed of the study's risks and benefits. No personal identifying information was recorded and respondents’ data were carefully uploaded on a passworded computer reserved for the study while being assured of the confidentiality of the information provided. The study methods were carried out in accordance with relevant guidelines and regulations.

**Consent for Publication**

Not applicable

**Availability of data and materials**

Datasets supporting the findings of this study are available on request to the corresponding author.

**Competing interests**

The authors declare that they have no competing interests.

**Funding**

The research work was self-sponsored as authors received no specific grant or any funding support from agencies in the public, commercial or not-for-profit sectors.

**Author contributions**

T.O.O contributed to the study conceptualization, methodology, analysis and wrote the final draft of the manuscript. B.P.O contributed to the study methodology, data collection and management, result analysis, discussion and first manuscript. B.A contributed to the methodology, analysis and revised the first draft of the manuscript. A.D.O contributed to the study methodology, data collection and management, and result analysis and revised the first draft of the manuscript. P.C.A contributed to the study conceptualization, methodology, data collection, production of the abstract and revised the first draft of the manuscript. I.S. contributed to the study methodology, and data collection and revised the first draft of the manuscript. All authors reviewed and approved the final draft of the manuscript.

**Acknowledgements**

The authors are grateful to final-year students of health-related disciplines in Obafemi Awolowo University, Ile-Ife for their cooperation during data collection for the study.

The first author is supported by the Consortium for Advanced Research Training in Africa (CARTA) which is funded by the Carnegie Corporation of New York (Grant No--B 8606.R02), SIDA (Grant No:54100029) and the Developing Excellence in Leadership, Training and Science in Africa (DELTAS Africa) Initiative (Grant No: 107768/Z/15/Z). The views expressed in this publication are those of the authors and not necessarily those of the partners in the consortium.

**REFERENCES**

1. Bundred P, Gibbs T. Medical migration and Africa: an unwanted legacy of educational change. Medical Teacher. 2007;29:893–6.

2. Akinwumi AF, Solomon OO, Ajayi PO, Ogunleye TS, Ilesanmi OA, Ajayi AO. Prevalence and pattern of migration intention of doctors undergoing training programmes in public tertiary hospitals in Ekiti State, Nigeria. Hum Resour Health. 2022;20:76.

3. Yamamoto TS, Sunguya BF, Shiao LW, Amiya RM, Saw YM, Jimba M. Migration of Health Workers in the Pacific Islands: A Bottleneck to Health Development. Asia Pac J Public Health. 2012;24:697–709.

4. Ahmat A, Okoroafor SC, Kazanga I, Asamani JA, Millogo JJS, Illou MMA, et al. The health workforce status in the WHO African Region: findings of a cross-sectional study. BMJ Global Health. 2022;7 Suppl 1:e008317.

5. Hagopian A, Thompson MJ, Fordyce M, Johnson KE, Hart LG. The migration of physicians from sub-Saharan Africa to the United States of America: measures of the African brain drain. Human Resources for Health. 2004;2:17.

6. World Health Organization. The World Health Report 2006: Working Together for Health. World Health Organization; 2006.

7. Ossai EN, Una AF, Onyenakazi1 RC, Nwonwu EU. Emigration Plans after Graduation of Clinical Medical Students of Ebonyi State University Abakaliki, Nigeria: Implications for Policy. Nigerian Journal of Clinical Practice. 2020;23:995–1003.

8. Chen PG, Nunez-Smith M, Berg D, Gozu A, Rulisa S, Curry LA. International medical graduates in the USA: a qualitative study on perceptions of physician migration. BMJ Open. 2011;1:e000138.

9. Murataj N, Syla B, Krasniqi Y, Bahtiri S, Bekaj D, Beqiri P, et al. Migration Intent of Health Care Workers during the COVID-19 Pandemic in Kosovo. International Journal of Environmental Research and Public Health. 2022;19:11122.

10. Silvestri DM, Blevins M, Afzal AR, Andrews B, Derbew M, Kaur S, et al. Medical and nursing students’ intentions to work abroad or in rural areas: a cross-sectional survey in Asia and Africa. Bull World Health Organ. 2014;92:750–9.

11. Clemens MA, Pettersson G. New data on African health professionals abroad. Human Resources for Health. 2008;6:1.

12. Amorha K, Irobi C, Udoh A. The brain drain potential of skilled health workers from sub-Saharan Africa: A case study of pharmacy students in Nigeria. Pharmacy Education. 2022;22:654–63.

13. Imran N, Azeem Z, Haider II, Amjad N, Bhatti MR. Brain Drain: Post Graduation Migration Intentions and the influencing factors among Medical Graduates from Lahore, Pakistan. BMC Research Notes. 2011;4:417.

14. Onah CK, Azuogu BN, Ochie CN, Akpa CO, Okeke KC, Okpunwa AO, et al. Physician emigration from Nigeria and the associated factors: the implications to safeguarding the Nigeria health system. Hum Resour Health. 2022;20:85.

15. Stilwell B, Diallo K, Zurn P, Vujicic M, Adams O, Poz MD. Migration of health-care workers from developing countries: strategic approaches to its management. Bulletin of the World Health Organization. 2004.

16. Adebayo A, Akinyemi OO. “What Are You Really Doing in This Country?”: Emigration Intentions of Nigerian Doctors and Their Policy Implications for Human Resource for Health Management. Int Migration & Integration. 2022;23:1377–96.

17. World Health Organization. Global strategy on human resources for health: workforce 2030. Geneva: World Health Organization; 2016.

18. Blacklock C, Ward AM, Heneghan C, Thompson M. Exploring the migration decisions of health workers and trainees from Africa: A meta-ethnographic synthesis. Social Science & Medicine. 2014;100:99–106.

19. Times Higher Education. Obafemi Awolowo University. Times Higher Education (THE). 2022. https://www.timeshighereducation.com/world-university-rankings/obafemi-awolowo-university. Accessed 21 Apr 2023.

20. Hosmer Jr DW, Lemeshow S, Sturdivant RX. Applied logistic regression. John Wiley & Sons; 2013.

21. Paul AM. Stepwise International Migration: A Multistage Migration Pattern for the Aspiring Migrant. American Journal of Sociology. 2011;116:1842–86.

22. Storz MA, Lederer A-K, Heymann EP. German-speaking medical students on international electives: an analysis of popular elective destinations and disciplines. Globalization and Health. 2021;17:90.

23. Saidi F. The metrics of the physician brain drain. Arch Iran Med. 2006;9:433–4.

24. Deressa W, Azazh A. Attitudes of undergraduate medical students of Addis Ababa University towards medical practice and migration, Ethiopia. BMC Med Educ. 2012;12:68.

25. Ebeye T, Lee H. Down the brain drain: a rapid review exploring physician emigration from West Africa. Global Health Research and Policy. 2023;8:23.

26. Toyin-Thomas P, Ikhurionan P, Omoyibo EE, Iwegim C, Ukueku AO, Okpere J, et al. Drivers of health workers’ migration, intention to migrate and non-migration from low/middle-income countries, 1970–2022: a systematic review. BMJ Glob Health. 2023;8:e012338.

27. Burch VC, McKinley D, Wyk J van, Kiguli-Walube S, Cameron D, Cilliers FJ, et al. Career intentions of medical students trained in six sub-Saharan African countries. Education for Health. 2011;24:614.

28. Ramos P, Alves H. Migration intentions among Portuguese junior doctors: Results from a survey. Health Policy. 2017;121:1208–14.

29. Suciu ŞM, Popescu CA, Ciumageanu MD, Buzoianu AD. Physician migration at its roots: a study on the emigration preferences and plans among medical students in Romania. Human Resources for Health. 2017;15:6.

30. Li W, Sun H. Migration intentions of Asian and African medical students educated in China: a cross-sectional study. Hum Resour Health. 2019;17:88.

31. Poudel C, Ramjan L, Everett B, Salamonson Y. Exploring migration intention of nursing students in Nepal: A mixed-methods study. Nurse Educ Pract. 2018;29:95–102.

32. Nguyen L, Ropers S, Nderitu E, Zuyderduin A, Luboga S, Hagopian A. Intent to migrate among nursing students in Uganda: Measures of the brain drain in the next generation of health professionals. Human Resources for Health. 2008;6:5.

33. World Health Organization. State of the world’s nursing 2020: investing in education, jobs and leadership. 2020.

34. Yeates N, Pillinger J. Human Resources for Health Migration: global policy responses, initiatives, and emerging issues. 2013. http://oro.open.ac.uk/39072/. Accessed 21 Apr 2023.

35. Simpson NB. Demographic and economic determinants of migration. IZA World of Labor. 2022. https://doi.org/10.15185/izawol.373.

36. Witt J. Addressing the migration of health professionals: the role of working conditions and educational placements. BMC Public Health. 2009;9:S7.
